# Italian good practice recommendations on management of persons with Long-COVID

**DOI:** 10.3389/fpubh.2023.1122141

**Published:** 2023-04-20

**Authors:** Marina Giuliano, Dorina Tiple, Piergiuseppe Agostoni, Benedetta Armocida, Ludovico Biardi, Anna Rita Bonfigli, Andrea Campana, Maria Ciardi, Fabiano Di Marco, Marco Floridia, Paola Gnerre, Tiziana Grassi, Ignazio Grattagliano, Paola Kruger, Matilde Leonardi, Rocco Liguori, Elisabetta Pagani, Elisa Perger, Flavia Pricci, Marinella Ruggeri, Andrea Silenzi, Francesco Spannella, Carlo Tascini, Giulia Teté, Matteo Tosato, Simona Vecchi, Marika Villa, Graziano Onder

**Affiliations:** ^1^National Center for Global Health, Istituto Superiore di Sanità, Rome, Italy; ^2^Department of Neuroscience, Istituto Superiore di Sanità, Rome, Italy; ^3^Heart Failure Unit, Centro Cardiologico Monzino IRCCS, Milan, Italy; ^4^Department of Clinical Sciences and Community Health, University of Milan, Milan, Italy; ^5^Department of Cardiovascular, Endocrine-Metabolic Diseases, and Aging, Istituto Superiore di Sanità, Rome, Italy; ^6^Healthcare Risk Management, IRCCS INRCA, Ancona, Italy; ^7^Scientific Direction, IRCCS INRCA, Ancona, Italy; ^8^Ospedale Pediatrico Bambino Gesù, Rome, Italy; ^9^Department of Public Health and Infectious Diseases, University “La Sapienza”, Rome, Italy; ^10^Department of Health Sciences and Pneumology, University of Milan, ASST Papa Giovanni XXIII, Bergamo, Italy; ^11^Department of Internal Medicine, ASL AL, Acqui Terme, Italy; ^12^SIMG, Italian College of General Practitioners and Primary Care, Florence, Italy; ^13^European Patients Academy for Therapeutic Innovation (EUPATI), Rome, Italy; ^14^Neurology, Public Health and Disability Unit, Fondazione IRCCS Istituto Neurologico Carlo Besta, Milan, Italy; ^15^UOC Clinica Neurologica, IRCCS Istituto delle Scienze Neurologiche, Bologna, Italy; ^16^Department of Biomedical and Neuromotor Sciences, University of Bologna, Bologna, Italy; ^17^Department of Internal Medicine, Fondazione IRCCS, Policlinico San Matteo, Pavia, Italy; ^18^Department of Cardiovascular, Neural and Metabolic Sciences, Sleep Disorders Center, Istituto Auxologico Italiano IRCCS, Milan, Italy; ^19^Provincial Health Service, Messina, Italy; ^20^General Directorate for Health Prevention, Ministry of Health, Rome, Italy; ^21^Internal Medicine and Geriatrics, IRCCS INRCA, Ancona, Italy; ^22^Department of Clinical and Molecular Sciences, Università Politecnica delle Marche, Ancona, Italy; ^23^Infectious Diseases Division, Department of Medicine, University of Udine and Azienda Sanitaria Universitaria Friuli Centrale, Udine, Italy; ^24^Department of Dentistry, IRCCS San Raffaele Hospital, Milan, Italy; ^25^Department of Geriatrics and Othopedic Sciences, Fondazione Policlinico Universitario "Agostino Gemelli" IRCCS, Rome, Italy; ^26^Department of Epidemiology, Lazio Regional Health Service, Rome, Italy; ^27^Department of Geriatrics, Università Cattolica del Sacro Cuore, Rome, Italy

**Keywords:** COVID-19, Long COVID, good clinical practices (GCP), Italy, health care systems, guideline

## Abstract

A significant number of people, following acute SARS-CoV-2 infection, report persistent symptoms or new symptoms that are sustained over time, often affecting different body systems. This condition, commonly referred to as Long-COVID, requires a complex clinical management. In Italy new health facilities specifically dedicated to the diagnosis and care of Long-COVID were implemented. However, the activity of these clinical centers is highly heterogeneous, with wide variation in the type of services provided, specialistic expertise and, ultimately, in the clinical care provided. Recommendations for a uniform management of Long-COVID were therefore needed. Professionals from different disciplines (including general practitioners, specialists in respiratory diseases, infectious diseases, internal medicine, geriatrics, cardiology, neurology, pediatrics, and odontostomatology) were invited to participate, together with a patient representative, in a multidisciplinary Panel appointed to draft Good Practices on clinical management of Long-COVID. The Panel, after extensive literature review, issued recommendations on 3 thematic areas: access to Long-COVID services, clinical evaluation, and organization of the services. The Panel highlighted the importance of providing integrated multidisciplinary care in the management of patients after SARS-CoV-2 infection, and agreed that a multidisciplinary service, one-stop clinic approach could avoid multiple referrals and reduce the number of appointments. In areas where multidisciplinary services are not available, services may be provided through integrated and coordinated primary, community, rehabilitation and mental health services. Management should be adapted according to the patient’s needs and should promptly address possible life-threatening complications. The present recommendations could provide guidance and support in standardizing the care provided to Long-COVID patients.

## Introduction

After more than 2 years from the beginning of the SARS-CoV-2 epidemic it is now widely recognized that for a significant number of people with the SARS-CoV-2 infection, the clinical manifestations are not limited to the acute symptomatic phase but can persist with a heterogeneous spectrum of subacute or chronic manifestations that prevent a complete return to the usual state of health. This condition of persistence of symptoms, that can affect subjects of any age and with different severity of the acute disease, has been recognized as a specific clinical entity, often referred to as Long-COVID. Long-COVID can affect multiple systems including cardiovascular, pulmonary, metabolic, coagulation and hematologic, renal, gastrointestinal, musculoskeletal, neurologic, and psychiatric disorders, with significant impacts on morbidity and mortality (1–4). A recent study also reported a significantly lower quality of life for Long-COVID patients compared to a healthy control group (5). Ongoing low-grade inflammation has been hypothesized to cause these symptoms, but the pathology remains largely unknown, knowledge on the best tools for its evaluation and diagnosis is still incomplete, and treatments are primarily based on symptoms relief.

Estimates of the proportion of persons who develop Long-COVID after acute infection are highly variable, ranging from less than 10% (6), to 20%–25% reported by the US Center for Disease Control and Prevention (7), to more than 50% reported in a large meta-analysis (8). Recently, the World Health Organization (WHO) estimated that approximately 10%–20% of COVID-19 patients experience lingering symptoms following an acute SARS-CoV-2 infection (9). Although it has been reported that both SARS-CoV-2 vaccination (10, 11) and the Omicron variant (12) are associated with a reduced incidence of the condition, given the hundreds of millions of cases of COVID-19 worldwide, a high number of patients is expected to suffer Long-COVID manifestations with a significant impact on health services.

Health systems have indeed been challenged to respond to the presence of this new condition, with the need to provide multidisciplinary care to a large number of patients presenting with an extreme clinical heterogeneity.

Specialized health services for individuals with Long-COVID have arisen in Italy to respond to the increasing need for supportive care but service organization for the management of this condition and the mode of service delivery are heterogeneous, with wide regional differences in terms of numbers and characteristics of referral centers for the diagnosis and care of the condition (13).

In 2021 a document elaborated by the Italian National Institute of Health provided interim guidance on general Long-COVID management principles (14), however, these indications needed to be updated and refined incorporating more recent knowledge on the topic.

### Aims

We sought to develop good practice recommendations for the clinical management of patients with Long-COVID in order to provide guidance and improve quality of care. These recommendations could contribute to standardize on a national basis the assistance provided - in terms of diagnosis, care and services organization - by the clinical centers and to strengthen their interactions.

The proposed recommendations could also contribute to optimize the use of health resources providing principles of priority of care based on the identification of subjects at higher risk of development of severe sequelae.

The proposed recommendations are targeted to the patients with Long-COVID, to healthcare professionals and the healthcare system.

## Methodology

### Definition of Long-COVID

Different definitions have been proposed for the condition. In line with the guidelines issued by the National Institute for Health and Care Excellence in UK (15) and by the Center for Disease Control and Prevention in the US (16), the panel agreed in defining Long-COVID as a syndrome characterized by signs and symptoms that persist or develop after >4 weeks after an acute SARS-CoV-2 infection. Although other definitions are used by other organizations, for example the WHO defines post-COVID as a condition occurring 3 months after acute infection, lasting for at least 2 months (17), the 4-week threshold allows a rapid identification and treatment of the consequences of the SARS-CoV-2 infection.

### Expert panel

The panel, representative of the multisystemic nature of Long-COVID, included specialists in respiratory diseases, infectious diseases and internal medicine, general practitioners, geriatricians, cardiologists, neurologists, pediatricians, and a dentist.

To take patient perspective into account, in terms of values, priorities and preferences, the panel also included a patient advocate.

The Panel met regularly on videoconferences, at least once per month between April and October 2022.

### Development of good practice statements

The process of development of the good practice statements (GPS) has followed, in part, the indications of the GRADE Working Group for definition of GPS (18, 19). The GPS do not derive from a systematic review of the literature but are based on experts’ consensus on different clinically important aspects for which supporting evidence is of low methodological quality or is not available. The choice of producing GPS rather than guidelines, is due to the lack of direct research evidence.

Initially, the research questions were collegially defined (Table 1). They included different areas of interest: access to Long-COVID services (questions 1 and 2), clinical evaluation (questions 3–5), and organization of services (questions 6 and 7).

**Table 1:** Research questions related to the management of Long-COVID patients that were defined by the expert panel.

**Access to Long-COVID services** 1. Who should be assessed for Long-COVID and when? 2. Who should evaluate the patient with a suspected condition of Long-COVID?
**Clinical evaluation** 2. What assessment should a patient with suspected Long-COVID receive? 3. When children should be evaluated for Long-COVID and what assessment should they receive? 5. How to assess Fatigue Respiratory symptoms (cough and dyspnea) Palpitations/tachycardia Chest pain Headache Autonomic dysfunction (gastrointestinal symptoms, hypotension, hyperhidrosis) Brain fog/cognitive impairment Anxiety/depression Sleep disorders Oral disorders Pain
**Organization of the services** 6. How should a patient with Long-COVID be managed? 7. How should a Long-COVID service be organized?

Then, a literature search was conducted for each research question (15, 16, 20–38). The search, conducted between April and September 2022, was performed on the main scientific databases (Pubmed, Medline) and on the scientific websites of the leading organizations in the fields of general medicine, infectious diseases, pneumology, neurology, cardiology and pediatrics. Predefined search terms included a combination of either “Long-COVID” or “Post-COVID” AND “guidelines” for the general research questions and a combination of either “Long-COVID” or “Post-COVID” AND “fatigue,” or “chest pain” etc. according to the specific research question. Main documents were selected and a summary of the available evidence for each research question was produced. The results of the literature search were then presented to the panel and discussed, and recommendations for each research question were formulated by members of the panel. Text controversies were defined by consensus. The final document of Good Practice Statements was finalized and approved by all the members of the panel.

The final document was also reviewed by three external reviewers. They provided comments that were analyzed by the members of the panel in a plenary session. Statements were modified according to the indications of the reviewers.

## Actionable recommendations

### Good practice statements

#### Question 1

*Who should be assessed for Long-COVID and when?*

- All patients hospitalized for COVID-19 should be evaluated for possible Long-COVID occurrence 4–6 weeks after discharge.
- Patients who were not hospitalized, who present new or persistent signs or symptoms not explained by an alternative diagnosis for >4 weeks after acute infection, should also be evaluated. Special attention should be posed to patients with frailty or clinical complexity who are at higher risk to develop typical or atypical complications of Long-COVID.
- Most commonly reported signs and symptoms to be considered, both as a single manifestation or more frequently in association, include the following: fatigue, cough, dyspnea, headache, sleep disturbances, cognitive impairment, loss of concentration, brain fog, anorexia, anosmia-dysosmia, ageusia-dysgeusia, myalgia, joint pain, anxiety, depression, palpitations, chest pain, sore throat, skin rash, gastrointestinal symptoms, xerostomia. Rare or atypical symptoms should not be overlooked especially in older patients and in children. Reduced performance at work or at school and reduced social interactions should also be not disregarded.
- Long-COVID is a diagnosis of exclusion that can be considered only after ruling out the complications due to diseases of other etiology.

#### Question 2

*Who should evaluate the patient with a suspected condition of Long-COVID?*

- Primary care providers (general practictioners and pediatricians) should be the first health professionals to evaluate patients with suspected Long-COVID.
- Care of patients with Long-COVID with a low level of clinical complexity can be coordinated and managed by the primary care providers.
- Patients with a high level of clinical complexity could be managed in a different context (i.e., hospitals or multidisciplinary services) but direct contact with the primary care provider of the patient should be maintained.
- In patients who have been hospitalized for COVID-19, an evaluation for symptoms of Long-COVID can be performed either by the primary care providers or in multidisciplinary services. Follow-up visits and diagnostic tests should be scheduled in a personalized and tailored manner according to the clinical needs of the patients.

#### Question 3

*What assessment should a patient with suspected Long-COVID receive?*

a. The first evaluation of a patient with suspected Long-COVID should include a detailed evaluation of the following:
  ο History of COVID-19 disease, nature, severity, timing of presentation of symptoms.
  ο Presence of signs and symptoms of Long-COVID including (but not limited to): fatigue, cough, dyspnea, headache, sleep disturbances, cognitive impairment, loss of concentration, brain fog, anorexia, anosmia-dysosmia, ageusia-dysgeusia, myalgia, joint pain, anxiety, depression, palpitations, chest pain, sore throat, skin rash, gastrointestinal symptoms, xerostomia. Rare or atypical symptoms should not be overlooked in older people and in children.
  ο Impact of symptoms on the activity of the individual (work/school), the level of autonomy, the functional status, the quality of life, and the social relationships.
  ο History of other health conditions and current therapy.
  ο Exacerbation of pre-existing conditions.

This information can be collected through questionnaires self-completed by patients.

b. A multidimensional evaluation should be performed in older people. This evaluation should consider, in addition to the above-mentioned issues, social isolation, functional status and falls, nutritional aspects, and cognitive symptoms, and could include the use of validated multidimensional screening tools.
c. The presence of a family member, especially for older people, could be important to help in describing symptoms and to have a complete clinical picture.
d. In patients with pre-existing health conditions, the impact of COVID-19 on these conditions should be evaluated and the current pharmacological therapy modified accordingly.
e. Laboratory tests, radiological and functional exams should be prescribed by the treating physician based on the signs and symptoms of the patient.
f. In patients with a history of severe COVID-19 disease or who have been hospitalized for COVID-19, the following tests should be prescribed:
  ο Blood tests: full blood count, renal and hepatic function tests, C-reactive protein, coagulation tests, Na/K/Ca/Mg. Glycosylated hemoglobin (HbA1c) should be prescribed in patients with a history of previous diabetes or suspected diabetes, thyroid hormones in patients with previous or suspected thyroid disease.
  ο Urinalysis

#### Question 4

*When children should be evaluated for Long-COVID and what assessment should they receive?*

- All children who have been hospitalized for COVID-19 should be evaluated 4–6 weeks after discharge.
- Children with clinical complexity should be re-evaluated after COVID-19 even if not hospitalized.
- Children, who have not been hospitalized, who have new or persisting signs or symptoms not explained by an alternative diagnosis, for >4 weeks after acute infection should also be evaluated.
- Signs and symptoms to be considered include those more prevalent in the Long-COVID syndrome in adults, but in children rare or atypical symptoms should not be overlooked.
- The first evaluation of a child with a suspected condition of Long-COVID should include a detailed evaluation of the following:
  ο History of COVID-19 disease, nature, severity, and timing of presentation of symptoms.
  ο Presence of signs and symptoms of Long-COVID, including those more prevalent and more rare and atypical symptoms.
  ο Impact of symptoms on school activity, learning ability, independence, quality of life, and social life.
  ο History of pre-existing health conditions and current therapy
  ο Exacerbation of pre-existing conditions.
- As during the pandemic a decrease in the coverage of recommended vaccinations has been observed, resulting in an increased risk of acquiring vaccine-preventable diseases in children, health professionals should continue to provide information on this issue to families, children and adolescents, including those with Long-COVID.
- Return to physical and sport activity of children and adolescents who had SARS-CoV-2 infection or who present Long-COVID symptoms should take place, after consultation with the primary care pediatrician, considering the type of physical activity and the severity of the COVID-19 disease.

#### Question 5 how to evaluate:

##### 5a. Fatigue

- Collect specific information on the onset of fatigue, on the presence of concomitant signs and symptoms and of emotional and psycho-social factors, on the use of potentially correlated drugs, on substance abuse, sleep disturbances, pre-existing conditions associated with chronic fatigue, organ-specific consequences of a severe COVID-19 infection.
- Measure clinical parameters such as blood pressure in both supine and standing positions, heart rate, breathing rate, body temperature and pulse oximetry at rest.
- If possible, perform *exercise tolerance test* according to the patient capacity (i.e., 1-min sit-to-stand test or 6-min walk test), monitoring oxygen saturation. These tests should be performed after a careful clinical evaluation and in line with existing guidelines.
- The cardiopulmonary exercise test, which represents the gold standard for the evaluation of exercise tolerance and the identification of the causes of fatigue, should be performed only after specialistic evaluation, according to the patient’s conditions and the availability of territorial services.
- Use standardized questionnaires such as *The Strengths and Difficulties Questionnaire* (*SDQ*), the *Fatigue Severity Scale (FSS)*, the *EuroQol 5-D* (*EQ-5D)*, and the *Post-Covid-19 Functional Status Scale (PCFS)* to assess the severity of fatigue over time.
- Perform blood tests (C-reactive protein, full blood count, renal function tests, TSH, Na/K/Ca/Mg, blood protein level, glucose); troponin and NT-proBNP/BNP can be considered if a cardiac origin is suspected (as for instance acute coronary syndrome or heart failure), and ECG, chest X ray and global spirometry can be performed based on the patient’s conditions.
- Consider the need of a rehabilitation program.
- Exclude the complications due to potentially life-threatening diseases, not related to COVID-19 infection.
- Further diagnostic tests should be prescribed by the treating physician according to patient’s clinical conditions and in agreement with the evidence that could emerge in the literature, given the possible fast advancements in the field.

##### 5b. Respiratory symptoms (cough and dyspnea)

- Collect clinical history on the onset of respiratory symptoms, their characteristics and the presence of concomitant symptoms.
- Assess vital signs (blood pressure, heart rate, breath rate, body temperature, pulse oximetry at rest).
- If possible, perform *exercise tolerance test* according to the patient capacity (i.e., 1-min sit-to-stand test or 6-min walk test), monitoring oxygen saturation. These tests should be performed after a careful clinical evaluation and in line with existing guidelines.
- The cardiopulmonary exercise test represents the gold standard for the evaluation of exercise tolerance and for the quantification and evaluation of the causes of dyspnea in cases of non-univocal interpretation. This test should be performed only after specialistic evaluation, according to the patient’s conditions and the availability of territorial services.
- Perform blood tests (C-reactive protein, full blood count, liver and renal function tests, TSH, Na/K, blood protein level, blood glucose). Determination of D-dimer can be performed if pulmonary embolism is suspected. If a cardiac origin is suspected, ECG, cardiac enzymes and NT-proBNP/BNP can be prescribed.
- Perform chest X-ray and respiratory function tests (spirometry with DLCO testing) in patients with new onset or persisting respiratory symptoms at ≥3 months after acute infection.
- Perform blood gas analysis only in the presence of reduced oxygen saturation at pulse oximetry at rest (< 95%) or during exercise tolerance tests.
- Perform high resolution chest CT scan without contrast medium 3–6 months after acute infection in patients with respiratory symptoms and abnormal respiratory function tests, to exclude other causes of dyspnea and to evaluate possible interstitial pulmonary disease.
- Perform transthoracic echocardiogram in patients with persisting symptoms suggestive of heart damage (chest pain, palpitations, signs, and symptoms of heart failure), in the presence of abnormal ECG or increased levels of NT-proBNP/BNP.
- Further diagnostic tests should be prescribed by the treating physician on the basis of the patient’s clinical conditions and in agreement with the evidence that could emerge in the literature, given the possible fast advancements in the field.

##### 5c. Palpitations/tachycardia

- For patients with palpitations/tachycardia the initial clinical evaluation should include a detailed clinical history, a physical examination, some basic blood tests (full blood count, TSH, renal function tests, Na/K/Mg/Ca), pulse oximetry and ECG; in case of clinically suspected cardiac involvement further tests (troponin, NT-proBNP/BNP) can be considered.
- An *exercise tolerance test*, according to patients’ capacity (6-min walk test), should be performed to evaluate possible cardiac deconditioning and the reduced tolerance to exercise. This test should be performed after careful clinical evaluation and according to existing guidelines.
- The cardiopulmonary exercise test, which represents the gold standard for the functional evaluation, should be performed only after specialistic evaluation, according to the patient’s conditions and the availability of territorial services.
- If a postural orthostatic tachycardia syndrome is suspected a “3-min active stand test” should be performed. This test should be performed after a careful clinical evaluation and according to existing guidelines.
- Further diagnostic exams (echocardiogram, ECG Holter, cardiac MRI, coronary CT angiogram) should be guided by the clinical history of the patient, by the physical examination, and by the results of the diagnostic tests.
- Self-monitoring can be considered if, according to the patient, is a possible option. Parameters to be monitored include heart rate, blood pressure, oximetry, and the presence of symptoms. It is important that patients are adequately instructed on how to perform the measurements, on how to interpret the results and understand when to contact the treating physician.

##### 5d. Chest pain

- Regardless of the possible correlation with Long-COVID, chest pain of suspected cardiac or pulmonary origin can be a manifestation of acute life-threatening conditions (acute coronary syndrome, aortic dissection, pulmonary embolism). In these cases, the patient should be quickly evaluated and referred to emergency services.
- For the diagnosis and management of chest pain it is recommended to follow existing guidelines and the diagnostic-therapeutic pathways already in use.

##### 5e. Headache

- The evaluation of patients with Long-COVID headache should include the specific clinical history (date of onset and main characteristics, pre-existing history of headache disorders and/or of neurological diseases), a general clinical evaluation (blood pressure, inspection and palpation of the temporal artery in patients >50 years, temporomandibular joint examination, cranial palpation of pain-trigger points and painful points) and a neurological examination (signs of meningeal irritation, gait disturbances, Romberg test, facial asymmetry).
- Some laboratory tests can be performed: full blood count, C-reactive protein, Na/K, renal function tests, thyroid hormones, total protein level. The measurement of peripheral blood oxygen can be considered.
- According to the patient’s conditions, neuroimaging studies can be considered to exclude a secondary headache if suspicious signs are reported in the medical history (the presence of treatment-resistant headache or of severe daily headache) and/or to the neurological exam, or in the presence of “red flags” such as an acute onset without previous episodes, the presence of attacks with new, different characteristics compared to the classical attacks, the onset after the age of 40 years.
- The management of a patient with Long-COVID headache should follow existing guidelines and the diagnostic-therapeutic pathways already in use for patients with non-COVID related headache disorders.

##### 5f. Autonomic dysfunction (gastrointestinal symptoms, hypotension, hyperhidrosis)

- Collect a clinical history including specific information on the onset of symptoms, psycho-social and emotional concomitant factors, the use of potentially correlated drugs, pre-existing conditions possibly associated to these disorders.
- Assess clinical parameters such as blood pressure and heart rate both in supine and standing positions, respiratory rate, body temperature, and pulse oximetry at rest.
- Use standardized questionnaires such as the Composite Symptom Scale 31 (COMPASS-31) to assess the severity of the symptoms and monitor them over time.
- A 3 or 10- min active stand test and a “Head-Up Tilt Table Test” should be performed in patients with symptoms of orthostatic intolerance such as dizziness, fatigue, or orthostatic headaches, to diagnose postural orthostatic tachycardia or orthostatic hypotension. These tests should be performed after a careful clinical evaluation and according to existing guidelines.
- The management of a patient with post-COVID autonomic dysfunction should not be different, in terms of diagnosis and treatment, from the management of dysautonomia not related to COVID-19.

##### 5g. Brain fog/cognitive impairment

- Perform a first-level evaluation of cognitive disorders in all patients older than 75 who have been hospitalized or who had a severe COVID-19 disease, and in all patients who report persistent cognitive disorders, “brain fog,” mental confusion and memory problems at least 4 weeks after the acute SARS CoV-2 infection.
- No tool for the evaluation of cognitive disorders has been studied specifically for the Long-COVID condition. Therefore, for the first-level assessment, simple and validated tools can be recommended: the Mini-Cog test, the Montreal Cognitive Assessment test (MoCA) and the Mini-Mental State Examination test (MMSE).
- Neuropsychological tests (second-level assessment) should be performed according to the patient’s clinical conditions limiting them to patients with clear cognitive impairment at the first-level tests.
- Clinical history, including a careful evaluation of pre-existing clinical conditions, should be collected in the presence of family members or caregivers.
- Further diagnostics exams should be guided by the clinical history of the patient, by the physical examination, and by the results of the diagnostic tests.

##### 5h. Anxiety and depression

- All hospitalized COVID-19 patients and patients with a history of pre-existing psychological or psychiatric disorders should be evaluated for the presence of psychological or psychiatric symptoms.
- To assess the severity of psychological or psychiatric symptoms and to monitor them over time the following questionnaires can be used: General Anxiety Disorder-7 (GAD-7), Patient Health Questionnaire-9 (PHQ-9), PTSD Checklist for DSM-5 (PCL-5), Impact of Event-Scale-Revised (IESR), Hospital Anxiety and Depression Scale (HADS), Hamilton-A (anxiety) and Hamilton-D (depression). In children the Multidimensional Anxiety Scale for Children (MASC-2), the Child Behavior Checklist (CBCL) and the Child Depression Inventory (CDI-2) can be used.
- In older people, in the presence of psychological or psychiatric symptoms, the coexistence of cognitive disorders should be evaluated using validated tests (see recommendations for “Brain fog”).
- The management of a patient with post-COVID anxiety or depression does not require a specific approach and therefore the diagnostic-therapeutic pathways already in use should be followed.

##### 5i. Sleep disorders

- To evaluate the severity of sleep disorders and to monitor them over time the following validated questionnaires can be used: the Pittsburg Sleep Quality Index or the Insomnia Severity Index for insomnia or the quality of sleep; the Berlin questionnaire and/or the Epworth Sleepiness Scale for daytime sleepiness. In children the Sleep Disturbance Scale for Children (SDSC) can be used.
- In case of excessive daytime sleepiness, the presence of sleep apnea should be excluded also using the questionnaires mentioned above (Berlin and/or Epworth Sleepiness Scale).
- The management of insomnia should include the evaluation and treatment of conditions of psychological or emotional stress, of correlated anxiety and/or depression, and the explanation of a correct sleep hygiene before performing further investigations or prescribing drugs.
- In the suspect of sleep apnea, a polysomnography or a nocturnal cardio-respiratory monitoring should be performed according to the availability of territorial services. If nocturnal events are detected the patient should be referred to specialized centers and the routine diagnostic-therapeutic process should be followed.
- The management of post-COVID sleep disorders should not be different, in terms of diagnosis and treatment, from that of the sleep disorders unrelated to COVID-19.

##### 5j. Oral disorders

- In the presence of oral disorders, a specialistic evaluation is recommended. This evaluation should include, besides the examination of the oral cavity, diagnostic tests to be defined on the basis of the clinical needs.
- Patients who have been hospitalized or who had a severe COVID-19 disease and have social or sanitary conditions of vulnerability should be evaluated by a dentist.
- Since during the pandemic there has been a reduction in the participation in the programs of dental health in the pediatric population, the Panel recommends that children of age below 14 years, and particularly those who have suffered of COVID-19, are included in the programs for the protection of dental health finalized to an early diagnosis.

##### 5k. Pain

- In the presence of pain, a specific clinical history should be collected with the evaluation of the date of onset, the type of pain, the location, the duration, the modifications with exercise or rest (factors that relieve, worsen or trigger it) and the response to analgesia. Pre-COVID-19 diseases, current co-existing symptoms (for instance depression symptoms) and the impact of pain on the functional state should be evaluated.
- In the presence of arthralgia/myalgia specific laboratory tests could include: full blood count, C-reactive protein, erythrocyte sedimentation rate, ferritin, uric acid levels, liver profile, renal profile, serum protein electrophoresis, muscle enzymes, rheumatoid factor, antinuclear antibodies. The prescription of these and of further tests (for instance anti-citrulline antibodies) should be based on the clinical conditions of the patient.
- In patients with suspected post-COVID neuropathy, the Panel recommends a specialistic evaluation to assess the need of specific diagnostic exams.
- A multidimensional evaluation is needed for the management of chronic pain and, if necessary, a multidisciplinary patient management for a targeted therapeutic pathway.

#### Question 6

*How should a patient with Long-COVID be managed?*

- After completing the evaluation for Long-COVID, an open discussion between the patient and the healthcare professional should be initiated, with the objective to define an individualized plan of care and a clinical and therapeutic tailored and personalized pathway for the patient’s specific needs through a shared decisional process.
- Patients should be informed on how to plan their gradual return to work (RTW), and, if needed, the plan for RTW should be shared with the occupational physician.
- Patients should receive information and training on self-management of Long-COVID symptoms including:
  ο how to self-manage symptoms, with realistic goals
  ο who to contact in case of worsening of symptoms or in the need of support
  ο how to obtain support from other services, as, for instance, integrated home health care, with family members/caregivers’ participation
- Patients should be invited to keep a registry/diary for the monitoring of the symptoms, their magnitude and evolution.
- The need of a multi-disciplinary rehabilitation including physical and neuro-cognitive aspects should be considered. The rehabilitation and management plan should be individualized through targeted rehabilitative trainings.
- Additional support for management should be directed to vulnerable subjects, as older people and the persons with complex needs. Additional support could be provided through integrated home health care programs or social services.
- A follow-up pathway that assures continuity of care should be carefully planned.

#### Question 7

*How should a Long-COVID care service be organized?*

- Long-COVID services should provide care pathways including multidisciplinary services, with multidisciplinary expertise. Both remote and in presence consultations should be available.
- In frail or older patients one-stop services (concentrating in 1 day different specialty consultations and diagnostic tests as, for instance, in Long-COVID day-hospital) should be preferred.
- For pediatric patients, services should work in a network to consider all the clinical, psychological, and social specificities in the different pediatric age groups.
- The presence of a case manager (responsible for the coordination of care of the patient) could also be important to guarantee an appropriate patient management, the continuity of assistance, the planning of interventions and the appropriate follow-up.
- Pathways should integrate local referral services, primary and community care, rehabilitation and specialty services, the multidisciplinary services, and the mental health specialistic services.
- A specific and continuous education on the Long-COVID conditions should be provided to all health professionals in the services caring for this condition.

## Conclusion

Long-COVID is a complex condition with heterogeneous presentation and relevant clinical impact, but specific pathways for diagnosis and care with a multidisciplinary approach are still to be defined.

Specialized health services for individuals with Long-COVID have arisen in Italy to respond to an increasing need for supportive and rehabilitative care. However, there was a need to provide recommendations to standardize the answer to specific needs as well as the quality of care on a national basis.

Professionals of different disciplines, reflecting the multidisciplinary nature of the condition, were invited by the National Institute of Health to participate in a Panel with the objective to issue recommendations on general health services organization and management of specific symptoms.

The Panel highlighted the importance of providing integrated multidisciplinary care in the management of patients after SARS-CoV-2 infection.

The Panel agreed that a multidisciplinary service (one-stop clinic) for assessment could avoid multiple referrals and help in reducing the number of appointments. In areas where multidisciplinary services are not available, services may be provided through integrated and coordinated primary, community, rehabilitation, and mental health services. Management should be adapted according to the patient’s needs and should start addressing promptly possible life-threatening complications.

The approach to the patient should then focus on specific symptoms or conditions with sharing care decision with patients. Prioritization of follow-up care may be considered for those at high risk for Long-COVID, including those who have been hospitalized and those more susceptible to complications (such as older adults and those with multiple comorbidities). A multidisciplinary rehabilitation program including physical and neuro-cognitive aspects should also be considered. Patients should be informed on how to plan their gradual return to work, and, if needed, the plan for return to work should be shared with the occupational physician, who may perform a specific assessment of fitness for work (39).

This document provides a basis to establish national standardized pathways of care and management of Long-COVID (in adults and children) by primary care providers, relevant specialists, mental health, and psychosocial professionals.

Fully understanding of Long-COVID is still incomplete. These recommendations are a living tool that will therefore be updated as new evidence emerges.

## Author contributions

MG and DT drafted the manuscript, PA, AB, AC, MC, FM, IG, PK, ML, RL, EPa, EPe, MR, CT, GT, and MT were members of the Panel issuing the recommendations. GO was the chair of the Panel. LB, FS, MG, and DT were responsible for the literature search. BA, TG, MF, FP, and MV provided substantial scientific contribution to finalize the manuscript. PG, AS, and SV critically revised the manuscript. All authors contributed to the article and approved the submitted version.

## Funding

The article was prepared in the framework of a project funded by the National Center for Diseases Prevention and Control of the Italian Ministry of Health (“Analysis and strategies of response to the long-term effects of COVID-19 infection (Long-COVID),” Grant ID: 6S19).

## Conflict of interest

The authors declare that the research was conducted in the absence of any commercial or financial relationships that could be construed as a potential conflict of interest.

## Publisher’s note

All claims expressed in this article are solely those of the authors and do not necessarily represent those of their affiliated organizations, or those of the publisher, the editors and the reviewers. Any product that may be evaluated in this article, or claim that may be made by its manufacturer, is not guaranteed or endorsed by the publisher.

## References

[1] Michelen M Manoharan L Elkheir N Cheng V Dagens A Hastie C. Characterising long COVID: a living systematic review. BMJ Glob Health. (2021) 6:e005427. doi: 10.1136/bmjh-2021-005427, PMID: 34580069 PMC8478580

[2] Davis HE Assaf GS McCorkell L Wei H Low RJ Reem Y. Characterizing long COVID in an international cohort: 7 months of symptoms and their impact. EClinicalMedicine. (2021) 38:101019. doi: 10.1016/j.eclinm.2021.101019, PMID: 34308300 PMC8280690

[3] Ayoubkhani D Khunti K Nafilyan V Maddox T Humberstone B Diamond I. Post-covid syndrome in individuals admitted to hospital with covid-19: retrospective cohort study. BMJ. (2022) 372:n693. doi: 10.1136/bmj.n693, PMID: 33789877 PMC8010267

[4] Donnelly GP Wang XQ Iwashyna TJ Prescott HC. Readmission and death after initial hospital discharge among patients with COVID-19 in a large multihospital system. JAMA. (2021) 325:304–6. doi: 10.1001/jama.2020.21465, PMID: 33315057 PMC7737131

[5] Líška D Liptaková E Babičová A Batalik L Bonárová PS Dobrodenková S. What is quality of life in patients with long COVID compared to a healthy control group. Front Public Health. (2022) 10:975992. doi: 10.3389/fpubh.2022.975992, PMID: 36408018 PMC9667067

[6] Global Burden of Disease Long COVID Collaborators. Estimated global proportions of individuals with persistent fatigue, cognitive, and respiratory symptoms clusters following symptomatic COVID-19 in 2020 and 2021. JAMA. (2022) 328:1604–15. doi: 10.1001/jama.2022.18931, PMID: 36215063 PMC9552043

[7] Bull-Otterson L Baca S Saydah BTK Adjei S Gray S. Post-COVID conditions among adult COVID-19 survivors aged 18-64 and ≥ 65 years – United States, march 2020-November 2021. MMWR Morb Mortality Wkly Rep. (2022) 71:713–7. doi: 10.15585/mmwr.mm7121e1

[8] Groff D Sun A Ssentongo AE Ba DM Parsons N Poudel GR. Short-term and long-term rates of postacute sequelae of SARS-CoV-2 infection a systematic review. JAMA Netw Open. (2021) 4:e2128568. doi: 10.1001/jamanetworkopen.2021.28568, PMID: 34643720 PMC8515212

[9] World Health Organization. (2022). Post COVID-19 condition. Available at: https://www.who.int/europe/news-room/fact-sheets/item/post-covid-19-condition (Accessed November 15, 2022).

[10] Al-Aly Z Bowe B Xie Y. Long COVID after breakthrough SARS CoV-2 infection. Nature Med. (2022) 28:1461–7. doi: 10.1038/s41591-022-01840-0, PMID: 35614233 PMC9307472

[11] Hastie CE Lowe DJ McAuley A Winter AJ Mills NL Black C. Outcomes among confirmed cases and a matched comparison group in the Long-COVID in Scotland study. Nat Commun. (2022) 13:5663. doi: 10.1038/s41467-022-33415-5, PMID: 36224173 PMC9556711

[12] Antonelli M Pujol JC Spector TD Ourselin S Steves CJ. Risk of long COVID associated with delta versus omicron variants of SARS-CoV-2. Lancet. (2022) 399:2263–4. doi: 10.1016/S0140-6736(22)00941-2, PMID: 35717982 PMC9212672

[13] Floridia M Grassi T Giuliano M Tiple D Pricci F Villa M. Characteristics of Long-COVID care centers in Italy. A national survey of 124 clinical sites. Front. Public Health. (2022) 10:975527. doi: 10.3389/fpubh.2022.975527, PMID: 36062113 PMC9437305

[14] Onder G Floridia M Giuliano M Lo Noce C Tiple D Bertinato L. (2021). Interim guidance on Long-COVID management principles. Version of July 1, 2021. Rome. Istituto Superiore di Sanità. Available at: http://www.iss.it/rapporti-iss-COVID-19-in-english (Accessed November 15, 2022)

[15] National Institute for Heath and Care Excellence. COVID-19 rapid guideline: managing the long-term effects of COVID-19. (2022). Available at: https://www.nice.org.uk/guidance/NG188 33555768

[16] Center for Disease Control and Prevention. Post-COVID conditions: Information for healthcare providers. (2022). Available at: https://www.cdc.gov/coronavirus/2019-ncov/hcp/clinical-care/post-covid-conditions.html

[17] World Health Organization. (2021). A clinical case definition of post COVID-19 condition by a Delphi consensus. Available at: https://www.who.int/publications/i/item/WHO-2019-nCoV-Post_COVID-19_condition-Clinical_case_definition-2021.1 10.1016/S1473-3099(21)00703-9 PMC8691845 34951953

[18] Guyatt GH Alonso-Coello P Schünemann HJ Djulbegovic B Nothacker M Lange S. Guideline panels should seldom make good practice statements: guidance from the GRADE working group. J Clin Epidemiol. (2016) 80:3–7. doi: 10.1016/j.jclinepi.2016.07.006, PMID: 27452192

[19] Lotfi T Hajizadeh A Moja L Akl EA Piggott T Kredo T. A taxonomy and framework for identifying and developing actionable statements in guidelines suggests avoiding informal recommendations. J Clin Epidemiol. (2022) 141:161–71. doi: 10.1016/j.jclinepi.2021.09.028, PMID: 34562579

[20] Yelin D Moschopolous CD Margalit I Gkrania-Klotsas E Landi F Stahl JP. ESCMID rapid guidelines for assessment and management of long COVID. Clin Microbiol Infect. (2022) 28:955–72. doi: 10.1016/j.cmi.2022.02.018, PMID: 35182760 PMC8849856

[21] Esposito S Principi N Azzari C Cardinale F Di Mauro G Galli L. Italian intersociety consensus on management of long covid in children. Ital J Pediatr. (2022) 48:42. doi: 10.1186/s13052-022-01233-6, PMID: 35264214 PMC8905554

[22] Sisó-Almirall A Brito-Zeron P Conangla Ferrin L Kostov B Moragas Monero A Mestres J. Long COVID-19: proposed primary care clinical guidelines for diagnosis and disease management. Int J Environ Res Public Health. (2021) 18:4350. doi: 10.3390/ijerph18084350, PMID: 33923972 PMC8073248

[23] American Thoracic Society. ATS statement: guidelines for the six-minutes walk test. Am J Respir Crit Care Med. (2002) 166:111–7. doi: 10.1164/rccm.166/1/111 12091180

[24] British Thoracic Society Guidance on Respiratory Follow Up of Patients with a Clinico-Radiological Diagnosis of COVID-19 Pneumonia V1.2 11 May 2020. (2020). Available at: https://www.brit-thoracic.org.uk/covid-19/covid-19-information-for-the-respiratory-community/

[25] Writing Committee Gluckman TJ Bhave NM Allen LA Chung EH Spatz ES. 2022 ACC expert consensus decision pathway on cardiovascular sequelae of COVID-19 in adults: myocarditis and other myocardial involvement, post-acute sequelae of SARS-CoV-2 infection, and return to play: a report of the American College of Cardiology Solution set Oversight Committee. J Am Coll Cardiol. (2022) 79:1717–56. doi: 10.1016/j.jacc.2022.02.003, PMID: 35307156 PMC8926109

[26] Raman B Bluemke DA Lüscher TF Neubauer S. Long COVID: post-acute sequelae of COVID-19 with a cardiovascular focus. Eur Heart J. (2022) 43:1157–72. doi: 10.1093/eurheartj/ehac031, PMID: 35176758 PMC8903393

[27] Tana C Bentivegna E Cho SJ Harriott AM Garcia-Azorin D Labastida-Ramirez A. Long COVID headache. J Headache Pain. (2022) 23:93. doi: 10.1186/s10194-022-01450-8, PMID: 35915417 PMC9340759

[28] Membrilla JA Caronna E Trigo-Lopez J Gonzales-Martinez A Layos-Romero A Pozo-Rosich P. Persistent headache after COVID-19: pathophysiology, clinic and treatment. Neurol Perspect. (2021) 1:S31–6. doi: 10.1016/j.neurop.2021.10.003 PMC8669731 38620971

[29] Larsen NW Stiles LE Miglis MG. Preparing for the long-haul: autonomic complications of COVID-19. Auton Neurosci. (2021) 235:102841. doi: 10.1016/j.autneu.2021.102841, PMID: 34265539 PMC8254396

[30] Buoite Stella A Furlanis G Frezza NA Valentinotti R Ajcevic M Manganotti P. Autonomic dysfunction in post-COVID patients with and without neurological symptoms: a prospective multidomain observational study. J Neurol. (2022) 269:587–96. doi: 10.1007/s00415-021-10735-y, PMID: 34386903 PMC8359764

[31] Bohannon RW Crouch R. 1-minute sit-to-stand test: systematic review of procedures, performance, and clinimetric properties. J Cardiopulm Rehab Prevent. (2019) 39:2–8. doi: 10.1097/HCR.0000000000000336 30489442

[32] Mehmet H Yang AWH Robinson SR. What is the optimal chair stand test protocol for older adults? A systematic review Disabil Rehabil. (2020) 42:2828–35. doi: 10.1080/09638288.2019.1575922, PMID: 30907166

[33] World Health Organization (2022). Clinical management of COVID-19. Living guideline. Avaialble at: https://www.who.int/publications/i/item/WHO-2019-nCoV-Clinical-2022.2 35917394

[34] Spruit MA Holland AE Singh SJ Tonia T Wilson KC Troosters T. COVID-19: interim guidance on rehabilitation in the hospital and post-hospital phase from a European Respiratory Society-and American Thoracic Society-coordinated international task force. Eur Respir J. (2020) 56:2002197. doi: 10.1183/13993003.02197-2020, PMID: 32817258 PMC7427118

[35] Gherlone EF Polizzi E Tetè G de Lorenzo R Magnaghi C Rovere Querini P. Frequent and persistent salivary gland ectasia and Oral disease after COVID-19. J Dent Res. (2021) 100:464–71. doi: 10.1177/0022034521997112, PMID: 33655804 PMC7930603

[36] Iranmanesh B Khalili M Amiri R Zartab H Aflatoonian M. Oral manifestations of COVID-19 disease: a review article. Dermatol Ther. (2021) 34:e14578. doi: 10.1111/dth.14578, PMID: 33236823 PMC7744903

[37] Attal N Martinez V Bouhassira D. Potential for increased prevalence of neuropathic pain after the COVID-19 pandemic. Pain Rep. (2021) 6:e884. doi: 10.1097/PR9.0000000000000884, PMID: 33537521 PMC7850724

[38] Eliaçik S Uysal Tan F Kocagül A. Post-COVID 19 and neuropathic pain. J Infect Dis Epidemiol. (2022) 8:247. doi: 10.23937/2474-3658/1510247

[39] Chirico F Nucera G Szarpak L Zaffina S. The cooperation between occupational and public health stakeholders and its decisive role in the battle against the COVID-19 pandemic. Disaster Med Public Health Prep. (2021) 17:1–2. doi: 10.1017/dmp.2021.375 PMC8814468 34937592

